# Dietary Arginine and Citrulline Supplements for Cardiovascular Health and Athletic Performance: A Narrative Review

**DOI:** 10.3390/nu15051268

**Published:** 2023-03-03

**Authors:** Hun-Young Park, Sung-Woo Kim, Jisoo Seo, Yanghoon P. Jung, Hyunji Kim, Ah-Jin Kim, Sonwoo Kim, Kiwon Lim

**Affiliations:** 1 Physical Activity and Performance Institute, Konkuk University, Seoul 05029, Republic of Korea; 2 Department of Sports Medicine and Science, Graduate School, Konkuk University, Seoul 05029, Republic of Korea; 3 CJ CheilJedang Food & Nutrition Tech, Jung-gu, Seoul 04527, Republic of Korea; 4 Department of Physical Education, Konkuk University, Seoul 05029, Republic of Korea

**Keywords:** L-Arginine, L-Citrulline, citrulline malate, cardiovascular health, athletic performance

## Abstract

The global market for nutritional supplements (NS) is growing rapidly, and the use of L-arginine (Arg), L-citrulline (Cit), and citrulline malate (CitMal) supplements has been shown to enhance cardiovascular health and athletic performance. Over the past decade, Arg, Cit, and CitMal supplements have received considerable attention from researchers in the field of exercise nutrition, who have investigated their potential effects on hemodynamic function, endothelial function, aerobic and anaerobic capacity, strength, power, and endurance. Previous studies were reviewed to determine the potential impact of Arg, Cit, and CitMal supplements on cardiovascular health and exercise performance. By synthesizing the existing literature, the study aimed to provide insight into the possible uses and limitations of these supplements for these purposes. The results showed that both recreational and trained athletes did not see improved physical performance or increased nitric oxide (NO) synthesis with 0.075 g or 6 g doses of Arg supplement per body weight. However, 2.4 to 6 g of Cit per day for 7 to 16 days of various NSs had a positive impact, increasing NO synthesis, enhancing athletic performance indicators, and reducing feelings of exertion. The effects of an 8 g acute dose of CitMal supplement were inconsistent, and more research is needed to determine its impact on muscle endurance performance. Based on the positive effects reported in previous studies, further testing is warranted in various populations that may benefit from nutritional supplements, including aerobic and anaerobic athletes, resistance-trained individuals, elderly people, and clinical populations, to determine the impact of different doses, timing of ingestion, and long-term and acute effects of Arg, Cit, and CitMal supplements on cardiovascular health and athletic performance.

## 1. Introduction

L-Arginine (Arg) and L-Citrulline (Cit) are amino acids (AA) that play important roles in the body, including in the production of nitric oxide (NO) and the removal of waste products during exercise [1]. The use of nutritional supplements (NS) for health promotion and athletic performance has become increasingly popular, and Arg and Cit are among the most commonly used supplements in these areas [1]. The global market for NS has shown continuous steady sales growth, with an estimated value of about $101.38 billion in 2018, rising to nearly $220.3 billion in 2020 and projected to reach $327.4 billion in 2030 [2,3]. Over half of adults consume NS daily, and industrial market regulations must be strengthened [4,5].

Functional drinks, and supplements that claim to enhance athletic performance, are popular among both recreational and elite athletes [6,7]. However, athletes should only consume products that are scientifically proven to be effective. Supplements with false or insufficient scientific evidence can negatively affect health and athletic performance, leading to doping problems for athletes [6,8]. Nevertheless, athletes continue to seek out convenient supplements to achieve outstanding performance in international and domestic competitions through athletic performance improvement [9].

The development and use of NS, which offers benefits such as disease prevention, improved performance, and recovery for athletes, has risen rapidly [10]. These products are often formulated in convenient forms, such as gels, bars, protein powders, pills, and beverages. Functional NS are a simple and effective means of quickly replenishing glucose, energy, and electrolytes during physical activity [11]. Existing functional NS have been scientifically verified for their benefits, such as those containing dietary fiber, vitamins, and probiotics [1]. Research on the effects of AA supplements is still ongoing [1].

This study used a narrative review method to evaluate the available literature on the effects of Arg and Cit supplementation on cardiovascular health and athletic performance. The researchers systematically searched multiple electronic databases, including PubMed, MEDLINE, and Scopus, for relevant studies published in English from 1 January 2010, to 10 November 2022. The search terms included “arginine”, “citrulline”, “citrulline malate”, “supplementation”, “cardiovascular health”, “hemodynamic function”, “endothelial function”, “anaerobic capacity”, “aerobic capacity”, “muscular strength”, “power”, “endurance performance”, and “athletic performance.” The inclusion criteria for the review were randomized controlled trials, systematic reviews, and meta-analyses that evaluated the effects of Arg and Cit supplementation on cardiovascular health and athletic performance in humans. The researchers analyzed and synthesized the findings from the selected studies to provide an overview of the effects of Arg and Cit supplementation on cardiovascular health and athletic performance. The review covered a range of outcomes, including endothelial function, blood pressure (BP), athletic performance, and muscle soreness. This narrative review method allowed the researchers to provide a comprehensive and descriptive summary of the available evidence on the effects of Arg and Cit supplementation on cardiovascular health and athletic performance.

## 2. Physiological Role of Arginine and Citrulline in Human

**Figure 1:**
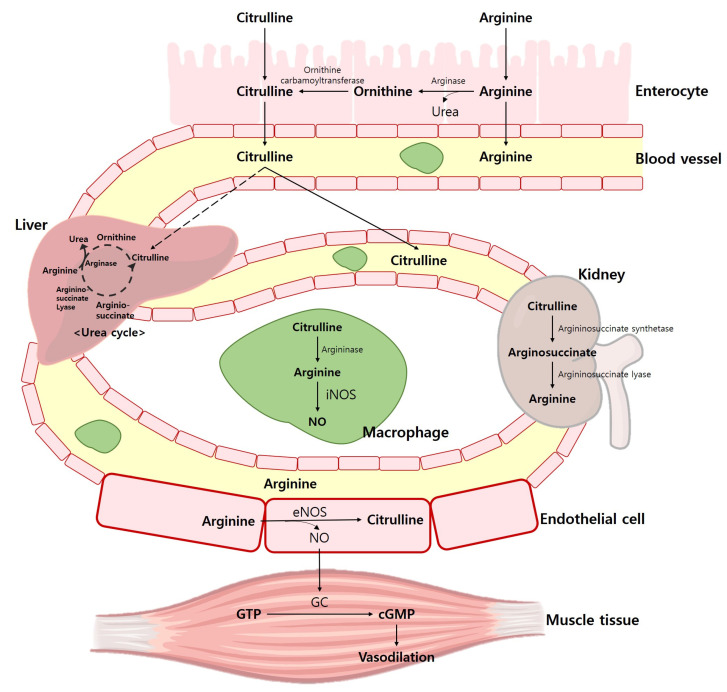
NOS through the Arg and Cit pathway. iNOS, cytokine-induced nitric oxide synthase; eNOS, endothelial nitric oxide synthase; NO, nitric oxide; GC, guanylate cyclase; GTP, guanosine triphosphate; cGMP, cyclic guanosine monophosphate.

Arg has been shown to enhance physical performance [12,13,14,15], vascular endothelium function [16,17,18,19], and sexual function [20,21]. Additionally, it is a conditionally essential AA that plays a role in various physiological processes. Therefore, a substantial amount of Arg is required to perform various physiological functions, and a significant amount of ingested Arg (~40%) is decomposed in the intestine while the remainder is transported to the liver where it is metabolized into urea [22,23,24]. Arg increases NO production and draws attention from athletes as having a potential ergogenic advantage [25], since Arg might be able to generate NO through nitric oxide synthase (NOS) [26]. The endogenous synthesis of NO occurs through a process in which Arg is metabolized into Cit by NOS [23]. Arg is also an important precursor for the production of NO. Argininosuccinate lyase and argininosuccinate synthase in the liver recycles Cit into Arg to produce a Cit-Arg cycle that produces NO [23]. The pathways of NOS include neural nitric oxide synthase (nNOS), cytokine-induced nitric oxide synthase (iNOS), and endothelial nitric oxide synthase (eNOS) [27]. In skeletal muscle, nNOS is the predominant isoform and regulates blood flow and glucose uptake during exercise. eNOS is primarily expressed in endothelial cells and produces nitric oxide in response to various stimuli, including physical activity. Details of NOS through the Arg pathway are shown in Figure 1.

**Figure 2:**
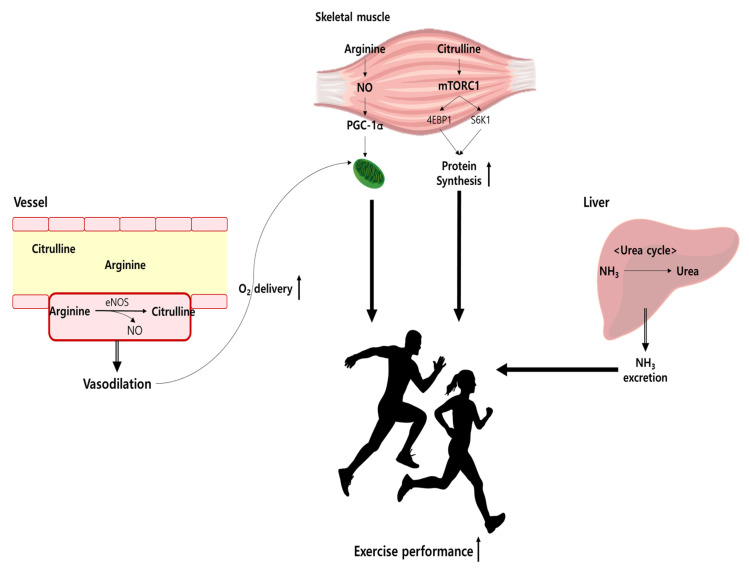
Arg and Cit pathway in exercise performance. eNOS, endothelial nitric oxide synthase; NO, nitric oxide.

NO acts on a variety of physiological processes, such as vasodilation, mitochondrial respiration, glucose absorption, and muscle contraction [28,29,30]. The roles NO plays in the human body are related to the improvement of exercise performance [30,31,32,33]. Therefore, nitrites and nitrates are generally used to quantify plasma NO concentrations, and it is most important to maintain physiologically appropriate NO concentrations for skeletal muscle health [34]. Figure 2 illustrates the Arg and Cit pathway in exercise performance.

### Intake of Arginine and Citrulline Supplements

Previous studies have reported that consuming three daily doses of 6 g/day of Arg along with small amounts of vitamins and branched-chain amino acids (BCAA), increased nitrite and plasma nitrate levels [32]. In addition, consuming Arg and other AA-rich NS, including Cit, BCAA, and fructose, decreased oxygen intake during moderate-intensity exercise, reduced plasma lactate production during exercise, and improved exercise tolerance during high-intensity exercise [32,35]. Taking an acute dose of 0.04 g/kg of Arg along with BCAA improved the sprint performance in handball athletes. However, consuming 6 g/day of Arg for three days did not improve intermittent anaerobic athletic performance in athletes [36]. Consuming 6 g of Arg for four weeks showed no change in hormone or metabolic parameters compared to exercise alone [37]. Both acute and chronic consumption of 5 g of Arg and two daily doses for 13 days were ineffective in improving cycling exercise performance in healthy young men [38]. Previous studies suggest that supplementing with Arg alone has limited impact on athletic performance, but combining it with other ingredients can improve exercise performance [39].

Cit is one of the non-essential AAs that can bypass liver metabolism to enhance Arg synthesis and improve NO bioavailability, as demonstrated in various studies [22,23,40,41]. Combined administration of Arg and Cit increases Arg concentration due to two AA synergies and improves NO biological availability [40,42]. The combination of Arg and Cit intake has been found to reduce energy consumption and enhance athletic performance more effectively than either Arg or Cit alone [42,43]. A study on elite taekwondo athletes who ingested Arg and Cit reported a reduction in exercise-induced central fatigue [44].

Interestingly, supplementation of Cit has been found to be more effective than Arg in increasing plasma Arg concentration [40,45,46]. Cit supplements can delay fatigue during high-intensity exercise by promoting ammonia removal and suppressing lactic acid accumulation in the blood through the urea cycle pathway [47]. Cit also improves the aerobic pathway by maintaining low plasma lactate concentrations [48]. However, the relationship between improved athletic performance and increased NO production in response to Cit supplementation is unclear [30]. Furthermore, previous studies that reported improved athletic performance with Cit supplementation used it in combination with malates and other components [30,49,50,51,52]. Most studies have reported that supplementation of Cit and malate is done in combination due to their synergistic coupling at the intramuscular level [52,53,54]. Malate, an intermediate product of the tricarboxylic acid cycle, can inhibit lactic acid production and increase energy production [52,55]. The efficacy of supplementing with citrulline malate (CitMal) cannot be defined solely as being related to Cit [1]. Further research is needed to determine the effectiveness of Cit independent of malate [56]. Previous studies evaluating the efficacy of Arg and Cit on NO biomarkers and athletic performance included BCAA or malate [39,53,56,57,58].

## 3. Effect of Cardiovascular Health

### 3.1. Hemodynamic Function

Arg is a substrate for numerous enzyme pathways including immune activation, vascular tone, and cell growth and is an AA obtained from dietary sources or produced endogenously [59]. In endothelial cells, Arg is metabolized to NO and Cit by NOS [59]. The positive effect of Arg supplementation on cardiometabolic markers, especially BP, insulin resistance, adiposity, and microvascular endothelial-dependent dilatation, has been well documented [60,61,62,63]. Studies have investigated the effect of Arg intake on resting BP in both healthy participants and mild hypertensive patients. The results showed that Arg-rich diets decreased systolic blood pressure (SBP) and diastolic blood pressure (DBP) [64]. In addition, daily supplementation with 3 to 12 g of Arg has been shown to prevent hypertension by reducing both SBP and DBP in mild hypertensive patients and in women with preeclampsia [65,66]. A meta-analysis showed decreased SBP and DBP in participants receiving 4–24 g/day (median: 9 g) over 2–24 weeks (median: 4 weeks) [67]. However, previous studies reported that Arg supplementation did not significantly change BP in mild chronic hypertensive pregnant women [68] and healthy young people [69]. This discrepancy may be due to various factors, including differences in participants, dosages, duration of intake, and BP ranges.

Cit is known to be an effective derivative of Arg that affects NO and cyclic guanosine monophosphate (cGMP) levels, but its effect on tissue perfusion in healthy subjects is not apparent [70,71,72,73]. Acute nitrate-Cit supplementation and placebo showed no difference in the post-ischemic vascular response measured by near-infrared spectroscopy in healthy young men [74]. However, other studies have reported an increase in muscle blood flow in healthy young participants after short-term (7 day) Cit supplementation during moderate-intensity exercise [73,75,76]. In healthy subjects, surrogate measurements of blood flow and endothelial function have not shown significant improvement, which may be due to physiological limitations of vascular compliance [72]. In addition, sympathetic nerve activation during exercise increases NO production in an eNOS-dependent manner in vascular endothelial cells, leading to local prioritization of systemic vasoconstriction in the arteries that supply active muscles [77]. These autoregulatory mechanisms may not be impacted by Cit supplementation in healthy individuals due to the intactness of the physiological process [78].

Cit, which is abundant in watermelons, is a non-essential AA [79] that can be metabolized to Arg. Arg is an essential AA that produces NO [79,80], which is responsible for its cardiac protective roles: from smooth muscle relaxation of blood vessels, induced by the NO-cGTP pathway to playing a crucial role in regulating BP [81]. Previous studies have reported that Cit supplementation increases the concentration of Arg in the human body and improves the biological availability of plasma Arg as a substrate for NO [54]. In a 6-week intervention study, supplementation with 6 g per day of watermelon extract containing both Cit and Arg led to a decrease in brachial and ankle SBP and DBP in pre-obese and hypertensive men, as well as a decrease in carotid augmentation index (AIx) [82]. A short-term intervention study (7–14 days) with 5.6 g of Cit supplementation per day reported a decrease in arterial stiffness in healthy, overweight middle-aged men [83,84]. A 6-week intervention study with 6 g per day of watermelon extract Cit supplementation in postmenopausal obese women receiving hypertension treatment, resulted in a decrease in arterial stiffness and aortic SBP, as well as a decrease in BP pulse wave reflection [85]. Watermelon-extract supplementation (Cit: 2.7 g/Arg: 1.3 g) was done for 6 weeks in middle-aged men and women with pre-hypertension, which led to a significant decrease in aortic SBP compared to the placebo controls, as well as a decrease in AIx and pulse wave velocity (PWV) [86]. In addition, previous studies have reported that Cit supplementation positively affects vascular wall stiffness measured by PWV and hypertension response to cold [82,83,84]. As some studies have proven the effect of reducing BP and arterial stiffness after Cit supplementation, but other studies have not demonstrated any effect, the impact of Cit supplementation on BP and arterial stiffness remains unclear [82,83,84,85,86,87,88,89,90,91]. The effects of Arg and Cit supplementation on hemodynamic function are summarized in detail in Table 1.

### 3.2. Endothelial Function

Cit indirectly increases NO biosynthesis by increasing Arg synthesis, which can improve the endothelial-mediated vasodilation function [71,72,94]. A decrease in the synthesis of NO has been linked to an essential role in endothelial dysfunction related to cardiometabolic diseases, menopause, and aging [95,96,97]. Previous studies in rodent models showed that reducing the biological availability of Arg resulted in increased microcirculation blood flow and NO synthesis when supplementing with Cit rather than Arg [98].

Arg, cGMP activity, and nitrate/nitrite (NO_x_) were improved in healthy young individuals when supplementing with Cit [70,71,99]. However, despite significant increases in Arg bioavailability and urinary NO_x_, there was no improvement in endothelial function as measured by brachial artery flow mediated dilatation (FMD) with acute or short-term supplementation (≤7 days) [71,72,94]. In the previous study, superficial femoral artery FMD was improved as a result of taking 10 g of Cit supplement daily for 4 weeks in hypertensive postmenopausal women [93]. In addition, intake of 10 g of Cit supplements daily for 4 weeks improved brachial artery FMD in hypertensive postmenopausal women [16]. Studies that supplemented 10 g of Cit in healthy young individuals showed increases in Arg and NO synthesis [72], indicating that the benefits of Cit supplementation may depend on the acute time period and the participants’ health status [78].

Endothelial dysfunction due to aging is associated with a decrease in NO synthesis and Arg bioavailability [95,97,100]. A study supplementing 10 g of Cit acutely in older adults with heart failure reported increased de novo Arg and NO synthesis, but at a lower synthesis rate compared to younger individuals [72]. Previous studies in elderly male subjects reported no changes in plasma NO_x_ or limb blood flow after rest and exercise and supplementing with Cit combined with whey protein alone or whey protein with other non-essential AA [94]. Even in healthy young participants, the intervention period may be an essential factor in determining the effect of FMD improvement from Cit supplementation. Previous studies found that eight weeks of supplementation with 800 mg of Cit per day was required to increase plasma Arg levels and improve FMD [101]. Additionally, 800 mg of Cit supplementation per day for eight weeks improves Arg/asymmetrical dimethylarginine levels and FMD in patients with vasospastic angina [101]. Studies on Arg supplementation and changes in FMD in response to Cit supplementation have shown similar results [16,93,102]. The variability in measurements in this field of study may be due to several factors, such as the relationship between the increase in the Arg/asymmetric dimethylarginine ratio and the improvement of FMD [78], or due to the reduction in NO production with increasing asymmetric dimethylarginine levels and aging-related endothelial dysfunction. Therefore, Cit or Arg replenishment may not improve endothelial function in the elderly [103].

Endothelial dysfunction caused by obesity-related insulin resistance is often mentioned as a major factor in the development of cardiovascular disease [96]. Different environmental factors such as fat cell-derived factors, high fat/high cholesterol diets, and aging contribute to the development of cardiovascular disease by exacerbating endothelial dysfunction and promoting low-grade inflammation [96,104,105,106,107]. The effects of supplementation of Arg and Cit on endothelial function are summarized in detail in Table 1.

## 4. Effect of Athletic Performance

### 4.1. Anaerobic and Aerobic Capacity

The supplementation of Arg has garnered attention for its potential to increase NO synthesis and improve athletic performance [58]. In earlier studies, male cyclists with training received either 0.075 g of Arg or a placebo per kg 60 min prior to completing a submaximal cycling exercise protocol [108]. Plasma metabolites were analyzed at various time points, including pre-supplementation (0 min), start of exercise (60 min), end of exercise (120 min), and end of rest (180 min) [108]. Results showed that the plasma Arg concentration of the supplement group significantly increased at all points from the start of exercise after Arg supplementation [108]. However, no significant difference was observed in plasma NO_x_ concentration in the Arg supplement group [108]. In a different study, elite male judo athletes were given 6 g per day of supplemental Arg for 3 days, which resulted in a significant increase in plasma Arg concentration after 60 min compared to baseline. However, there was no significant difference in plasma NO_x_ concentration compared to the placebo group after 60 min [36]. A study using an acute 6 g of Arg supplementation in resistance-trained physical education students reported no significant change in plasma nitrate concentration from pre-supplementation values till 60 min after exercise [109]. Other previous studies reported a tendency for plasma nitrite concentration to increase after acute intake of Arg supplementation at the same time points of 0 to 90 min, but no significant change was observed [110].

A study of 6 g of acute Arg supplementation in healthy male participants reported no significant difference in plasma NO markers at all points [111]. Similarly, studies of oral administration of 6 g of Arg or placebo daily for 1 month in healthy postmenopausal women reported no significant change in plasma NO synthesis [112]. The findings of previous studies indicate that a 6 g dose of Arg supplementation did not effectively increase NO levels. Recent meta-analysis studies have suggested that higher Arg supplement doses may be more effective [58], although a study of 10 g of Arg supplementation in male non-professional participants reported no significant change in plasma NO_x_ concentrations [113]. In a study examining plasma Arg levels in active young men, both low and high doses of acute Arg supplementation reported similar effects and no effect on NO synthesis [114]. Studies of continuous 6 g of Arg supplementation daily for 4 weeks in trained runners reported insufficient evidence of significant increase in NO synthesis [37]. The ability to improve and maintain NO synthesis, which plays a vital role in vasodilation, is necessary for increased oxygen uptake in skeletal muscle [115]. The limited effect of Arg supplementation on NO synthesis may be associated with Arg metabolism. Depleted plasma Arg levels may fail to maintain NO synthesis [1]. Approximately 15% of Arg is metabolized in the liver and 60% in the gastrointestinal tract, and increased NO production through oral ingestion of Arg may be impaired [116]. Some previous studies have suggested that sheer vascular stress is considered a major stimulus for endothelial NO synthesis during exercise in healthy participants, and therefore Arg supplementation may be unnecessary [111]. However, Arg supplementation may be beneficial for those with endothelial dysfunction, which affects NO synthesis in participants with atherosclerosis risk factors [111]. Other previous studies reported that taking Arg supplements may not continuously improve muscle blood flow and endothelial function during exercise [69]. On the other hand, some studies have reported positive results, such as improved cardiac performance in moderate congestive heart failure patients [117].

The results of previous studies on the effects of Arg supplementation on athletic performance are mixed. In a study of wrestling elite athletes, one dose of 1.5 g of Arg supplement or placebo per 10 kg body weight was found to increase the time exhausted during incremental cycle ergometer testing compared to the placebo group, but there were no significant differences in heart rate and oxygen consumption [118]. Contrastingly, a study of male soccer players who consumed 2 g of Arg supplements per kg of body weight for 45 days reported an improvement in maximum oxygen consumption [92]. Curiously, previous studies that reported significant improvements did not measure plasma concentrations of Arg, NO_x_, and mechanism studies that reported improvements in athletic performance were not clear [1].

Additionally, studies have shown that combining Arg supplementation with BCAA, aspartic acid, or other AA may improve aerobic capacity in healthy participants [32,119,120]. Yet, in a study of aerobically trained cyclists consuming 0.075 g of Arg supplement per kg of body weight 60 min before submaximal cycling exercise, there was no significant difference in oxygen consumption, heart rate, SBP, and DBP, which were measured at the start and end of the 60 min cycling protocol [108]. Other previous studies reported no significant change in the time to exercise duration and the steady-state pulmonary oxygen uptake during moderate-intensity exercise after ingesting a 6 g of Arg supplement beverage [110]. In addition, chronic intake of Arg supplements did not result in improved athletic performance in well-trained endurance athletes [121]. Furthermore, a study of healthy young men taking 5 g of Arg or 5.5 g of dextrin twice a day for 13 days found no significant difference in mean power output during cycling performance [38]. Therefore, the available evidence suggests that oral Arg supplementation in healthy participants is ineffective in improving the physiological response associated with improving aerobic capacity and increasing NO synthesis.

Citrulline supplements are commonly available in three different forms: standalone Cit, CitMal, and watermelon juice [53]. Oral Cit supplementation increases NO synthesis by elevating plasma Arg concentration [122], and if Arg availability is limited, Cit supplementation can restore NO production [71]. Clinical studies examining the effects of acute or chronic Cit supplementation in several chronic patients, including those with heart failure, obesity, and arteriosclerosis, reported positive effects on NO_x_ levels [123,124], however, inconsistent results were found in healthy participants [58]. In previous studies, the increase in blood NO_x_ concentration following Cit supplement ingestion varied based on the dose and duration of intake [73,101]. In a study where recreationally active participants ingested 3.4 g of Cit per day from watermelon juice for 16 days, there was a significant increase in plasma nitrite and plasma Arg [73]. In a study of male collegiate track athletes who took 3 g of Cit supplements daily for seven days, plasma NO_x_ concentrations were higher compared to baseline [125]. However, in most previous studies, Cit supplements were ineffective in changing NO biomarkers. A study comparing the effect on NO biomarkers after taking 6 g of Cit, Arg or placebo supplements per day for seven days in recreationally active male participants [75] showed that the plasma Cit and Arg concentrations were significantly increased after Cit supplementation, but plasma nitrite concentrations were not significantly increased [75]. In a study of 2.4 g of Cit supplementation per day for eight days in healthy trained men, plasma Arg concentration increased significantly following a cycling exercise protocol, but there was no difference in plasma NO_x_ concentration [126]. Additionally, there was no significant difference in plasma NO observed in a study of swimmers who took 8 g of Cit, Arg or placebo supplements per day for eight days [127]. In a study conducted with 3 or 9 g of Cit supplementation and submaximal exercise protocols in recreationally active participants, plasma NO_x_ concentrations were reported to be significantly reduced [128].

Ingestion of 6 g of Cit before completing a graded exercise test on a treadmill showed no significant change in maximum oxygen consumption or exhaustion time [129]. Acute intake of 6 g of Cit supplements in healthy and trained athletes may be insufficient to improve aerobic or anaerobic performance [53,129]. In addition, a study of 8 g of Cit supplementation for eight days in swimmers did not result in a reduction of 100 m or 200 m swimming trial times [127]. However, long-term supplementation may be more effective than acute intake of Cit supplements [39]. In a study where 3.4 g of Cit was consumed daily for 16 days in the form of watermelon juice concentrate, there was no difference in exhaustion time during a high-intensity exercise test despite an increase in plasma nitrite concentration [73].

A cycling performance evaluation in a study of 6 g of Cit supplementation per day for seven days in trained cyclists resulted in improved time trial performance, average power output, heart rate, and rate of perceived exertion (RPE) [130]. In a study of 6 g of Cit supplementation per day in participants of recreational activities, the mean arterial pressure and oxygen consumption mean response time were reduced, and total workload and tolerance to high-intensity of exercise were significantly improved [75]. As an AA intermediate product, Cit is known to play an essential role in the urea cycle, reducing ammonia toxicity in muscles [1]. In a study of male collegiate track athletes taking 3 g of Cit supplements daily for 7 days, RPE decreased, and oxygen consumption and average power output increased after performing intermittent short-time high-intensity protocols [125]. In a study of healthy trained men supplementing with 2.4 g of Cit per day for eight days, cycle ergometer trial completion times were reduced, average power output was increased, and subjective concentration and fatigue were also improved [126]. Cit supplements improve the aerobic pathway, lower plasma lactic acid concentrations during half marathon races, and alleviate post-race muscle soreness [48]. Decreased glycogen use in muscles during exercise leads to decreased plasma lactic acid and less reliance on anaerobic glycolysis in the energy metabolic system [47]. Supplementation with Cit enhances oxidative production of adenosine triphosphate (ATP) by inhibiting ammonia levels and glycolysis and preventing activation of phosphofructokinase, an important indicator of anaerobic glycolysis [48]. Detailed summaries of previous studies on the effects of Arg, Cit, and CitMal supplementation on anaerobic and aerobic capacity can be found in Table 2.

### 4.2. Muscular Strength, Power, and Endurance Performances

The intake of standalone Arg supplements by well-trained athletes or healthy recreational participants does not appear to improve strength [39]. Forbes et al., conducted a study in which strength-trained males ingested 0.075 g of Arg supplements or placebo per kg of body weight 60 min prior to performing a resistance exercise protocol. The results showed an increase in growth hormone response over time, but no difference in RPE [131]. Additionally, researchers found no significant difference in isokinetic knee extension performance or bench press when resistance-trained physical education students consumed either 6 g of Arg supplement or placebo [109]. However, a study reported a significant reduction in peak torque for elbow flexion and extension after resistance exercise following the ingestion of 3 g of Arg supplementation by physically active male and female participants [136]. The ineffectiveness of Arg supplements may be related to the phenomenon of blunted growth hormone responses after resistance exercise, which resistance exercise alone can stimulate [140,141]. Arg supplements can inhibit endogenous hormones that inhibit growth hormone release and increase hormones that promote growth hormone secretion and insulin-like growth factor-1 [142,143]. However, oral administration of Arg supplements has not been shown to increase exercise-induced growth hormones [144]. In addition, a study of male bodybuilders reported a decrease in growth hormone response with specific AA intake [145]. However, a study of trained runners who took Arg supplements continuously for 4 weeks suggested an increase in growth hormone production [37]. A recent meta-analysis study reported that long-term intake of approximately 1.5 to 2 g of Arg per day can improve aerobic and anaerobic performance [58]. A study of long-term Arg supplementation in intensity-trained men found that it could improve maximal bench presses [13]. It is important to note that previous studies reporting the effectiveness of long-term Arg supplement intake also included other active ingredients, such as alpha-ketoglutaric acid, ornithine, and aspartic acid [119,121,143,146]. Most of the studies that reported significant improvement after taking Arg supplements contained additional compounds [147]. In addition, studies that investigated the consumption 6 g of Arg supplementation per day for acute or prolonged periods reported no benefit for muscle strength, endurance, or maximum number of repetitions [148].

Cit supplements may help alleviate fatigue when taken at a dose of 3 to 4 g 60 min prior to exercise [57]. A recent meta-analysis suggested that acute Cit supplementation can enhance power performance and high-intensity strength [56]. However, most previous studies have focused on the impact of combining Cit with malate [149]. Additionally, many studies have only investigated the effect of Cit supplementation on resistance exercise performance in individuals who meet pre-determined inclusion criteria [1]. In a study where athletes completed one repetition maximum (1RM), taking 6 g of Cit supplements and a sucrose mixture 60 and 120 min before performing a graded exercise test on a treadmill did not significantly improve exercise performance or change the number of repetitions during a chest press test [129]. Acute Cit supplementation alone has also been shown to not improve high-intensity exercise performance in previous studies [54]. A study that administered 2 g of Cit per day for 8 weeks along with strength training programs to resistance-trained male participants did not result in a significant improvement in bench press performance [123]. In the previous study, leg curl strength was improved as a result of taking 10 g of Cit supplement daily for 4 weeks and slow velocity low-intensity resistance training in hypertensive postmenopausal women [93]. In addition, intake of 10 g of Cit supplements daily for 12 weeks and high-intensity interval training improved upper limbs muscle strength and walking speed in dynapenic-obese elderly [139]. This lack of improvement may be due to the fact that Cit is not as effective on its own as when ingested with malate [1]. Malate, which helps reduce lactic acid production and increases ATP production during high-intensity anaerobic exercise, may improve athletic performance [51].

According to a review study, taking a single acute dose of 8 g of CitMal 1 h prior to exercise in resistance-trained men and women may improve dynamic muscle endurance and strength performance [53]. In a study involving resistance-trained men, the iteration of failure was improved in all sets of barbell bench presses (except for the first two sets) after ingestion of 8 g of CitMal 1 h before exercise [50]. Similar results were observed in a study of resistance-trained females, with improved performance responses during six sets of plate-loaded leg press and bench press exercises at 80% 1RM [132]. In addition, previous studies have also reported that taking 8 g of CitMal 1 h before exercise led to an increase in the number of repetitions to failure during bodyweight exercises, hack squats, and leg press at 60% 1RM [51,52]. The effect size of these results was insignificant, suggesting that CitMal supplementation effectiveness may be weaker in these scenarios. Nevertheless, small improvements in effect over multiple sessions could enhance training adaptation through training programs [149].

The efficacy of CitMal supplementation on resistance exercise was assessed through the implementation of the German volume training (GVT) protocol, which involved consuming 8 g of CitMal 1 h before exercise [137,150]. Results indicated that CitMal supplementation did not affect the number of repetitions to failures during isokinetic dynamometer leg curls or barbell curls. Furthermore, there was no significant change in maximal isometric, eccentric and concentric force [137]. The lack of performance improvement may be attributed to disproportionate ratios of Cit and malate in the CitMal compounds used, as well as the training methods and dosages employed. Some previous studies have failed to find a positive effect of CitMal supplementation on isokinetic or dynamic muscular power indices [56,133,135,138]. A study of resistance-trained males who consumed 8 g of CitMal prior to a 40-min resistance exercise session (five sets of up to 15 repetitions of barbell bench press exercises at 75% 1RM) reported no change in mean power, peak power, fatigue index, or total repetitions performed [133]. Another previous study found that 8 g of CitMal taken 2 h before exercise had no effect on peak torque, average torque, total work, or metabolic efficiency, blood flow, or lactic acid clearance during five sets of concentric leg extensions [56].

Studies reporting the potential to support the adaptation characteristics and muscle function recovery of human muscle structures following the intake of CitMal supplements remain limited [123,134]. A study examining the effects of 6 g of CitMal supplementation before exercise found no significant impact on muscle damage markers, lower extremity muscle endurance recovery, or electromyography activation at 24, 48, and 72 h after exercise [134]. Long-term supplementation of one of 2 g CitMal, 2 g Cit, 200 mg glutathione, and a placebo over 8 weeks had no effect on maximal muscle strength but showed a large effect on reducing fat mass [123]. The limited impact on performance and adaptation may be due to the reduced dose of CitMal used. However, CitMal supplementation has shown promise in reducing fat mass and helping to manage athlete body composition. Further research is needed to determine the long-term effects of CitMal intake, as well as to identify optimal dosages, timing, and duration for enhancing athletic performance and body composition. The effects of supplementation of Arg, Cit and CitMal on muscular strength, power, and endurance performance found in previous studies are summarized in detail in Table 2.

## 5. Conclusions and Future Prospects

Taken together, the results of studies conducted on both recreational athletes and trained athletes show that supplementing with 0.075 g or 6 g of Arg per kg body weight did not enhance physical performance and perceptual feeling of exercise or increase NO synthesis. In addition, consuming 2.4 to 6 g of Cit per day for 7 to 16 days of various NSs increased NO synthesis, improved athletic performance, and reduced feelings of exertion. The results of acute supplementation with 8 g of CitMal are inconsistent but may increase muscle endurance, warranting further investigation. Given the positive outcomes reported in prior studies, it is recommended to conduct additional testing on the impact of various forms of Arg, Cit, and CitMal supplementation, including acute and long-term or loading doses, and varying timing of ingestion, on cardiovascular health and athletic performance in various populations (i.e., aerobic and anaerobic athletes, resistance-trained individuals, the elderly, and clinical populations) that may benefit from NS. Furthermore, studies comparing the effects of Cit and CitMal are limited. It is unclear whether malate, Cit, or both, are responsible for the effects observed in studies on CitMal supplementation. Recent research has shown that exercise can have a positive effect on the gut microbiota [151]. For example, studies have found that regular exercise can increase the diversity and abundance of beneficial bacteria in the gut, while also reducing the number of harmful bacteria. This can lead to improved gut health, a stronger immune system, and better overall health outcomes. Future studies should continue to investigate the potential synergistic effects of Arg, Cit, and CitMal supplements on various factors such as body composition, cardiovascular function, cognitive function, and muscle quality.

## Figures and Tables

**Table 1:** The effects of Arg and Cit supplementation on cardiovascular health.

Study	Participants	BP Status	Formulation	Dose	Duration	Results
Pahlavani et al. (2017) [92]	52 healthy males (Arg: 21.32 ± 4.59 y pla: 20.40 ± 4.04 y)	Normotensive	L-Arginine	2 g/day	45 days	FBS, TG, LDL, Cholesterol ↓ HDL ↑ SBP, DBP ↔
Suzuki et al. (2019) [42]	24 male soccer players (19.0 ± 0.2 y)	Normotensive	Arg + Cit	both 1.2 g/day each	6 days	Plasma post-exercise NO_x_, Cit, Arg ↑ Perception of physical exertion ↑
Figueroa et al. (2010) [91]	17 healthy young males (22 ± 1 y)	Normotensive	L-Citrulline	6 g/day	4 weeks	bSBP, aSBP, aPP ↓
Figueroa et al. (2016) [90]	16 overweight/obese healthy males (24 ± 2 y)	Normotensive	L-Citrulline	6 g/day	2 weeks	aSBP, aPP, AIx during IHG ↓ aDBP, MAP, AIx during PEMI ↓ aSBP, DBP, aPP, an baPWV during PEMI + CPT ↓ (resting) Attenuated the increase in aSBP and AIx during IHG and reduced MAP aDBP
Figueroa et al. (2013) [85]	12 postmenopausal women (57 ± 1 y)	Hypertensive	Watermelon Extract	6 g/day	6 weeks	(Resting) baPWV, aSBP, aDBP, aSBP2 ↓ (Resting) Alx ↔
Figueroa et al. (2011) [86]	9 middle-aged adults (4 M, 5 F) (54 ± 3 y)	Pre-hypertensive	Watermelon Extract	2.7 g/day	6 weeks	(Resting) bPP, aSBP, aPP, Alx ↓
Figueroa et al. (2012) [82]	14 middle-aged adults (3 M, 11 F) (58 ± 1 y)	Pre-hypertensive	Watermelon Extract	2.7 g/day	6 weeks	(Resting) ankle SBP, DBP, MAP ↓ (Resting) bSBP, bDBP, bMAP ↓ (Resting) carotid Alx ↓
Massa et al. (2016) [87]	40 prehypertensive and hypertensive (21 M, 19 F) (Cit: 48.7 ± 1, Pla: 47.4 ± 1)	Pre-hypertensive	Watermelon Extract	6 g/day	6 weeks	(Resting) bSBP and bDBP ↓ (Resting) cardiac autonomic function ↔
Maharaj et al. (2022) [16]	25 postmenopausal women (14 Cit, 11 Pla) (Cit: 61 ± 6 y, Pla: 64 ± 6 y)	Hypertensive	L-Citrulline	10 g/day	4 weeks	Serum L-ARG levels and brachial artery FMD ↑ (Resting) aortic DBP and MAP ↓
Kang et al. (2023) [93]	24 postmenopausal women (13 Cit, 11 Pla) (Cit: 62 ± 2 y, Pla: 63 ± 1 y)	Hypertensive	L-Citrulline	10 g/day	4 weeks	Superficial femoral artery FMD ↑

Abbreviations. FBS, fasting blood sugar; TG, triglyceride; LDL, low density lipoprotein; HDL, high density liprotein; SBP, systolic blood pressure; DBP, diastolic blood pressure; MAP, mean arterial pressure; NO_x_, NO metabolites; Cit, citrulline; Arg, arginine; NO, nitric oxide; bSBP, brachial systolic pressure; aSBP, aortic systolic blood pressure; aPP, aortic pulse pressure; bPP, brachial pulse pressure; aDBP, aortic diastolic blood pressure; bDBP, brachial diastolic blood pressure; Alx, augmentation pressure index; IHG, isometric handgrip exercise; PEMI, post-exercise muscle ischaemia; CPT, cold pressor test; baPWV, brachial-ankle pulse wave velocity; Pla, placebo; FMD, flow-mediated dilation.

**Table 2:** The effects of Arg, Cit, and CitMal supplementation on athletic performance.

Study	Participants	Exercise	Exercise Protocol	BP Status	Supplement Timing	Formulation	Dose	Duration	Results
Bailey et al. (2010) [32]	9 healthy males (26.0 ± 6.0 y)	aerobic exercise (cycle MIE, HIE)	MIE intensity: 80% GET HIE intensity: (power output at the VO_2_peak—power output at the GET) × 70% + power output at the GET Supplement 1 day: 2 × 6 min MIE, supplement 2 day: 1 × 6 min MIE + 1 × 6 min HIE, supplement 3 day: 1 × 6 min MIE + HIE until no longer able to perform the exercise	Normotensive	1 h before exercise	L-Arginine	6 g	Cross-over acute	Plasma NO_2_ ↑, SBP ↓ (MIE) VO_2_ ↑ (HIE) VO_2_ slow component amplitude ↓, Extended the time to exhaustion
Bailey et al. (2015) [75]	10 healthy adult males (19.0 ± 1.0 y)	aerobic exercise (cycle MIE, HIE)	During the first laboratory visit, subjects completed a ramp incremental cycle test on an electronically braking cycle ergometer (Lode Excalibur Sport, Groningen, The Netherlands). Initially, subjects performed 3 min of baseline cycling at 0 W; then the work rate was increased by 30 W/min until the limit of tolerance. Subjects rode their bikes at their own pace (70–90 rpm).	Normotensive	60 min before exercise	L-Arginine L-Citrulline	Arg 6 g/day or Cit 6 g/day	Cross-over 7 days	(Cit) BP ↓, VO_2_ kinetic, endurance exercise performance ↑
Bailey et al. (2016) [73]	8 healthy, recreationally active males (22 ± 2 y)	-	3 min of baseline cycling at 20 W. A passive recovery of 5 min separated the transitions. The moderate-intensity steps were each of 4 min. First visit and on day 14 of the period, they cycled at a severe-intensity constant-work-rate (70% Δ) until exhaustion. On day 16 of the period, subjects cycled for 6 min at a severe-intensity constant-work-rate (70% Δ) followed immediately by a 30 s all-out effort. The resistance on the pedals was set using the linear mode of the Lode ergometer so that the subjects would attain the power output calculated to be 50% Δ if they attained their preferred cadence.	Normotensive	75 min before test	Watermelon juice	~3.4 g/day	2 weeks	(resting) aSBP and MAP ↑
Vanhatalo et al. (2013) [110]	18 males recreationally active (22 ± 3 y)	aerobic exercise (treadmill)	Ramp incremental running tests on a motorized treadmill	Normotensive	95 min before exercise protocol	L-Arginine	6 g	Acute	NO_x_ and O_2_ cost of exercise or exercise tolerance ↔
Bailey et al. (2015) [75]	10 males recreationally active (19 ± 1 y)	aerobic exercise	Cycle ergometer; 3 “step” exercise tests: 2 moderate-intensity step tests followed by 1 severe-intensity exercise bout. Moderate-intensity step tests were completed to assess VO_2_ economy in the absence of a VO_2_ kinetics and cycling intensity step tests were completed to assess VO_2_ presence of a VO_2_ slow component	Normotensive	90 min before exercise protocol	L-Citrulline or L-Arginine	Cit 6 g/day Arg 6 g/day	7 days	L-Arg for both L-Arg and L-Cit ↑, Nitrite for L-Arg ↑, Mean arterial pressure ↓, Tolerance during severe exercise ↑, Lowered the VO_2_ mean response time ↓, Total amount of work completed in the exercise performance test with L-Cit supple but not with L-Arg ↑
Bailey et al. (2016) [73]	8 males recreationally active (22 ± 2 y)	aerobic exercise	‘Step’ exercise tests including one moderate-intensity step test followed by one high-intensity exercise bout	Normotensive	75 min before exercise protocol	Watermelon juice	300 mL/day (provided ~3.4 g/day L-Cit)		L-Arg, L-Cit, nitrite, skeletal muscle oxygenation index during moderate-intensity exercise ↑, Resting blood pressure ↑ Time-to-exhaustion during severe-intensity exercise ↔
Pahlavani et al. (2017) [92]	56 male soccer players (20.85 ± 4.29 y)	aerobic exercise (Harvard Step Test)	Harvard Step Test: repeated steps 30 times per 1 min using 50 cm platform, total of 5 min	Normotensive	evening	L-Arginine	2 g/day	45 days	VO_2_max ↑ BMI, BFM, LBM ↔
Forbes et al. (2013) [108]	15 male trained cyclists (28 ± 5 y)	aerobic exercise (cycle)	VO_2_max test on cycle ergometer—graded, incremental exercise to volitional exhaustion	Normotensive	60 min before exercise protocol	L-Arginine	0.075 g/kg	Acute	L-Arg ↑, NO_x_, GH, cardiorespiratory parameter measured ↔
Yavuz et al. (2014) [118]	9 male trained wrestlers (24.7 ± 3.8 y)	aerobic exercise (cycle)	Maximal incremental exercise on cycle ergometer starting at 60–70 rpm (increase by 30 watts at every 3 min)	Normotensive	60 min before exercise	L-Arginine	1.5 g/10kg	Acute	Time to exhaustion ↑, Lactate ↔
Suzuki et al. (2019) [42]	20 male soccer players (19.0 ± 0.2 y)	aerobic exercise	PWC 75% HRmax, three stages of load (25, 75 and 125 W) for 3 min each (total, 9 min)	Normotensive	60 min before exercise protocol	L-Arginine and L-Citrulline	Arg 1.2 g and Cit 1.2 g/day	7 days	Power output, L-Cit, L-Arg, NO_x_ ↑, RPE ↓
Pérez-Guisado and Jakeman (2010) [50]	41 healthy males (29.8 ± 7.64 y)	anaerobic exercise	The pectoral workout protocol comprised 16 sets in the following order: 4 sets of flat barbell bench presses (80% 1RM weight for the flat bench press), 4 sets of incline barbell bench presses (80% 1RM weight for the flat bench press), 4 sets of incline flies (60% 1RM weight for the flat bench press), and 4 sets of flat barbell bench presses (80% 1RM weight for the flat bench press). The speed of each rep was 3-4 s Rest for 1 min between sets and for 2 min between each exercise. The training program was the same during the 2-week study period	Normotensive	60 min before test	Citrulline malate	8 g	Cross-over acute	The number of repetitions ↑ Muscle soreness ↓
Mor et al. (2018) [43]	28 male active football players (18–30 y)	anaerobic exercise (RAST)	Running anaerobic sprint test (RAST): 6 × 35 m sprints at 10 s rest	Normotensive	3 g before training (1 g before breakfast and 2 g 30 min before training) and 3 g after training (2 g 1 h after training and 1 g before sleep) rest day 3 g (2 g before breakfast and 1 g before sleep)	L-Arginine	6 g or 3 g	14 days	BMI, recovery HR, AST, ALT, LDH ↓ anaerobic performance ↔ rapid reduction recovery LA
Terasawa and Nakada (2019) [125]	9 male track athletes (20.9 ± 1.6 y)	anaerobic exercise	Wingate test, using cycle ergometer, was adopted as the intermittent short-time high intensity exercise	Normotensive	60 min before exercise protocol	L-Citrulline	3 g/day	7 days	L-Cit group; ↓ RPE, ↔ Lactate, ↑ NO_x_, ↑ mean power output, ↑ pedaling speed, ↑ VO_2_
Sanchez-Gonzalez et al. (2013) [84]	16 young, healthy male adults (23 ± 3 y)	isometric handgrip (IHG) exercise		Normotensive		L-Citrulline	100 mg/kg	2 weeks	CI and IHG increases in bSBP, aSBP and Alx ↓
Forbes et al. (2014) [131]	14 males resistance trained (25 ± 4 y)	resistance exercise	Resistance exercise (3 sets of 8 exercises, 10 repetitions at ~75% 1RM)	Normotensive	60 min before exercise protocol	L-Arginine	0.075 g/kg	Acute	L-Arg ↑, GH ↓, RPE ↔
Meirelles and Matsuura (2018) [109]	12 males resistance trained (27 ± 3 y)	resistance exercise	Maximal dynamic strength in the bench press and knee extension (one-repetition maximum [1RM] test)	Normotensive	60 min before exercise protocol	L-Arginine	6 g	Acute	NO_x_, in strength exercises ↔
Bendahan et al. (2002) [55]	18 sedentary males symptomatic of fatigue (31 ± 9 y)	resistance exercise	Finger flexions performed at 1.5 s intervals lifting a 6 kg weight for 3 min Performed: 2 × before ingestion CM 3 × during ingestion CM 1 × after ingestion CM	Normotensive		Citrulline Malate	6 g/day	15 days	Power (w) ↑ Delta change in pH per unit of power ↓, Rate of oxidative ATP production (%EC) ↑, Rate of PCr resynthesis (mmol/min) ↑
Pérez-Guisado and Jakeman (2010) [50]	41 resistance trained males (30 ±8 y)	resistance exercise	4 sets at 80% 1RM until failure before and again after a pectoral training workout 1-min rest between sets, Barbell bench press	Normotensive	1 h before test	Citrulline Malate	8 g	Acute	Bench press total reps ↑, Total reps ↑ Muscle soreness following 24h, 48h ↓
Wax et al. (2015) [52]	12 resistance trained males (22 ±1 y)	resistance exercise	5 sets at 60% 1RM until failure 3-min rest between sets, Leg press, hack squat, leg extension	Normotensive	1 h before test	Citrulline Malate	8 g	Acute	Leg press total reps ↑, Hack squat total reps ↑, Leg extension total reps ↑ Blood lactate, HR, SBP, DBP ↔
Wax et al. (2016) [51]	14 resistance trained males (23 ±2 y)	resistance exercise	3 sets until failure 3-min rest between sets, Chin-up, Reverse chin-up, Push-up	Normotensive	1 h before test	Citrulline Malate	8 g	Acute	Chin-up total reps ↑, Reverse chin-up total reps ↑, Push-up total reps ↑ Blood lactate, HR, SBP ↔, DBP ↓
Glenn et al. (2017) [132]	15 resistance trained females (23± 3 y)	resistance exercise	6 sets at 80% 1RM until failure 1-min rest between sets, Bench press, Leg press	Normotensive	1 h before test	Citrulline Malate	8 g	Acute	Bench press total reps ↑, Leg press total reps ↑ Bench press RPE ↓ Leg press RPE, bench press HR, leg press HR ↔
Gonzalez et al. (2018) [133]	12 recreational resistance trained males (21 ± 2 y)	resistance exercise	5 sets × 15 reps at 75% 1RM 2-min rest between sets, Barbell bench press	Normotensive	40 min before test	Citrulline Malate	8 g	Acute	Total reps, peak power, mean power, fatigue index ↔ RPE, muscle thickness, subjective feelings of focus, energy, fatigue, and muscle pump ↔
da Silva et al. (2017) [134]	9 recreational active males (24 ± 3 y)	resistance exercise	1 set at 100% of 10RM machine leg press machine hack squat	Normotensive	1 h before test	Citrulline Malate	6 g	Acute	Total no. Reps ↔ RPE, lactate, creatine kinase, muscle soreness, testosterone-cortisol, electromyography ↔
Trexler et al. (2019) [135]	27 recreationally active males (22 ± 4 y)	resistance exercise	Maximal concentric leg extensions 5 sets × 30 reps	Normotensive	2 h before test	Citrulline Malate	8 g	Acute	NO_x_, blood flow, metabolic efficiency, hormonal response ↔
Hwang et al. (2018) [123]	75 resistance trained males (21 ± 2 y)	resistance exercise	1RM testing: free weight bench press angled leg press performed at baseline, 4, and 8 weeks of training	Normotensive		Citrulline Malate	2 g/day	8 weeks	Bench press 4 weeks, 8 weeks ↔, Angled leg press 4 weeks, 8 weeks ↔ Body mass, fat mass, body water ↔, Lean mass ↑ at week 4
Stanelle et al. (2020) [130]	10 male cyclists (24 ± 3 y)	compound	Simulated 40-km TT on a cycle ergometer, and supramaximal sprint repeat task (six 1-min sprints at 120% of maximal power)	Normotensive	120 min before exercise protocol	L-Citrulline	6 g/day	7 days	TT time ↔, average power output, HR and RPE ↑
Glenn et al. (2017) [132]	15 females (23.0 ± 3.0 y)	resistance exercise	1RM measurement (plate-loaded, flat barbell bench press and a plate-loaded, leg press)	Normotensive	60 min before exercise	Citrulline Malate	8 g	Acute	Upper-body repetitions, lower-body total repetitions ↑ upper-body RPE ↓, HR ↔
Esen et al. (2022) [127]	15 trained/developmental (5 females) swimmers and triathletes (25.0 ± 7.0 y)	200 m and 100 m freestyle swimming	Swimming Time Trials protocol (200 m and 100 m). 10 min after the warm-up, participants completed a 200 m freestyle TT. The participants recovered in a seated position for 30 min and were only allowed to drink water, which was recorded and precisely replicated on the 2nd trial. After 30 min recovery, a 100 m TT was performed. All TTs were started from a diving box and timed with a stopwatch.	Normotensive		L-Arginine or L-Citrulline	8 g/day	8 days	NO_x_, 200 m and 100 m swimming, BLa ↔
Cutrufello et al. (2015) [129]	Mixed athletes (11 M, 11 F) (20.6 ± 1.2 y)	aerobic exercise	Chest press; maximum number of repetitions at 80% 1RM for 5 sets with a 30 s rest period between each set. Bruce protocol on treadmill	Normotensive	60 and 120 min before exercise protocol	L-Citrulline	6 g	Acute	Number of repetitions, time to exhaustion, VO_2_max ↔
Streeter et al. (2019) [136]	30 healthy, physically active participants (20.4 ± 1.8 y) (15 M, 15 F)	acute resistance exercise (isokinetic dynamometer)	5 × 10 maximal isokinetic extension repetitions of the elbow joint at 90°	Normotensive	55 min before exercise	L-Arginine	3 g	Acute	Elbow extension and flexion, FMD, BP, HRV ↔
Chappell et al. (2018) [137]	15 recreational resistance trained adults (11 M, 4 F) (24 ± 2 y)	resistance exercise	10 sets × 10 reps 70% of Concentric force max 1-min rest between sets, Leg curl – knee extensor and flexor strength	Normotensive	1 h before test	Citrulline Malate	8 g	Acute	Total Reps, Isometric force max, Concentric force max, Eccentric force max ↔ Blood lactate ↔, Quadriceps muscle soreness ↑
Farney et al. (2019) [138]	12 recreationally trained adults (6 M, 6 F) (24 ± 4 y)	resistance exercise	1 set × 15 reps at 180° sec, Leg extension	Normotensive	1 h before test	Citrulline Malate	8 g	Acute	Total Reps, Peak torque, Peak power, Fatigue index ↔ Lactate, Heart rate ↔
Wong et al. (2016) [89]	41 postmenopausal overweight or obese females (WBVT + pla: 58.0 ± 4.0 y, Cit: 58.0 ± 4.0 y, WBVT + Cit: 58.0 ± 3.0 y)	whole-body vibration training	Consisted of static and dynamic squats with a 90° knee angle (considering 180° as full knee extension), semi-squats with 120° knee angle, wide-stance semi-squats, and calf raises. The training volume was increased progressively over the 8-week training period by increasing the intensity of vibration (25–40 Hz of frequency and 1–2 mm of amplitude), duration of the exercise set (30–60 s), number of sets (1–5), and total duration of the training session (11–60 min) and decreasing the duration of rest periods (60–30 s) between sets.	Normotensive	before breakfast and before sleeping	L-Citrulline	6 g/day	–8 weeks	AP, Brachial and aortic BP, BP, Alx, Alx@75 ↓ NO_x_ ↑
Buckinx et al. (2018) [139]	56 dynapenic-obese elderly (26 Cit, 30 Pla) (Cit: 65.7 ± 4.2 y, Pla: 68.1 ± 4.2 y)	high-intensity interval training	30 min exercise session 5 min warm-up at low intensity (50–60% maximal heart rate and/or a score between 8 and 12 on the Borg scale) 20-min HIIT of multiples 30 s sprints at a high intensity (80–85% maximal heart rate or Borg’ scale > 17) alternating with sprints of 90 s at a moderate intensity (65% maximal heart rate or Borg’scale score 13–16) 5 min cool-down (50–60% maximal heart rate and/or a Borg’ scale score 8–12)	Normotensive	every day during lunch meals	L-Citrulline	10 g/day	12 weeks	upper limbs muscle strength ↑ walking speed ↑
Kang et al. (2022) [93]	24 postmenopausal women (13 Cit, 11 Pla) (Cit: 62 ± 2 y, Pla: 63 ± 1 y)	slow velocity low-intensity resistance training	slow velocity low-intensity resistance training 4 lower body exercises (leg press, leg extension, leg curl, and calf raise) lasting approximately 25 min per session, 3 times a week Intensity: 40% and 50% of the estimated 1RM for the first and second 2 weeks All exercise movements with a slow speed contraction (3 s concentric and 3 s eccentric) using a metronome for 3 sets of 15 repetitions with 1–3 min of rest between sets	Hypertensive	a daily dose of 10 g taking 6 pills in the morning and 7 at night	L-Citrulline	10 g/day	4 weeks	leg lean mass and curl strength ↑

Abbreviations. MIE, moderate-intensity exercise; HIE, high-intensity exercise; NO_2_, nitrogen dioxide; O_2_hb, oxyhemoglobin; VE, ventilation; MAP, mean arterial pressure; O_2_, oxygen gas; CO_2_, carbon dioxide; BMI, body mass index; BFM, body fat mass; LBM, lean body mass; RPE, rate perceived exertion; VO_2_max, maximal oxygen consumption; AST, aspartate aminotransferase; ALT, alanine aminotransferase; LA, lactate acid; CI, cold induced; IHG, intermittent hand grip exercise; REP, repetition each press; pH, potential of hydrogen; ATP, adenosine triphosphate; PCr, phosphocreatine; NO_x_, NO metabolites; BLA, blood lactate concentrations; FMD, flow-mediated dilation; LDH, lactate dehydrogenase; 1RM, one Repetition Maximum.

## Data Availability

Not applicable.
